# Goal-setting improves movement accuracy during unsupervised training in stroke patients

**DOI:** 10.1038/s41598-025-17709-4

**Published:** 2025-10-03

**Authors:** Chikako Sakakibara, Kazuaki Oyake, Yukie Abe, Takashi Shigematsu, Tomoya Kitade, Ichiro Fujishima, Satoshi Tanaka

**Affiliations:** 1 Department of Rehabilitation Medicine, Hamamatsu City Rehabilitation Hospital, Shizuoka, Japan; 2 Department of Physical Therapy, School of Health Sciences, Shinshu University, Nagano, Japan; 3 Department of Psychology, Hamamatsu University School of Medicine, 1-20-1 Handayama, Chuo-ku, Hamamatsu, 431-3192 Shizuoka Japan

**Keywords:** Goal-setting, Motivation, Self-rehabilitation, Stroke, Unsupervised training, Rehabilitation, Neurology, Psychology

## Abstract

This pilot study investigated whether goal-setting leads to good behavioral performance and high motivation during an unsupervised reaching task in patients with subacute stroke. Using a single-blind, stratified randomized controlled design, 50 patients with unilateral upper-extremity motor paralysis were allocated to either a goal-setting group (*n* = 25) or a control group (*n* = 25) without goal setting. Both groups performed an unsupervised reaching task during a 15-minute session. The primary outcome measures included the total time spent in training and the success rate of reaching tasks. The secondary outcome was self-reported motivation assessed using a visual analog scale. Although there were no significant differences in the median [interquartile range] training time between goal-setting and control groups (636 [500–709] sec vs. 633 [494–715] sec, *p* = 0.771), the goal-setting group demonstrated significantly higher success rates than the control group (92.6 [82.9–96.0]% vs. 80.7 [65.4–92.6]%, *p* = 0.019). Self-reported motivation scores showed no significant differences between the groups (76 [60–95] vs. 84 [49–97], *p* = 0.994). These findings suggest that goal setting can help patients accurately perform reaching movements during unsupervised training, potentially enhancing the quality of motor performance in self-rehabilitation.

**Supplementary Information:**

The online version contains supplementary material available at 10.1038/s41598-025-17709-4.

## Introduction

Stroke is a leading cause of disability worldwide, and its increasing incidence has led to a growing demand for effective and extended rehabilitation^1,2^. Following a stroke, research suggests that people who do larger amounts of practice achieve better activity outcomes^3,4^. Self-rehabilitation programs, where patients perform exercises independently with minimal clinical supervision, have emerged as a promising approach to increase the amount of rehabilitation practice while reducing healthcare burden^5^. Recent studies and meta-analyses have demonstrated that self-rehabilitation programs can be as effective as conventional therapy in enhancing motor function and activity following stroke^5–7^.

One potential approach to enhance the effectiveness of self-rehabilitation is goal setting^5,8,9^. Goal setting is widely recognized as an integral part of rehabilitation^10,11^ and has been recommended in national clinical guidelines for stroke^12^. Several theories underpin goal setting in rehabilitation^11^ including Bandura’s Social Cognitive Theory, which emphasizes the role of observational learning and self-efficacy^13^ and Locke and Latham’s Goal Setting Theory^14^ which focuses on goal specificity and difficulty.

Studies have demonstrated that goal setting can significantly improve motor task performance in various patient populations^15,16^. In stroke rehabilitation, several studies and systematic reviews have suggested the positive effects of goal setting on patients’ psychological outcomes such as self-efficacy or motivation to rehabilitation^17–23^. Previous research has demonstrated that goal setting can enhance stroke patients’ perceptions of self-care ability^24^ which could potentially improve their participation in unsupervised training activities. Although these findings suggest that goal setting could enhance performance and motivation in self-rehabilitation after stroke, there is limited evidence examining its effects on patient engagement and motor performance during unsupervised training, particularly during the early stages of rehabilitation when patients are still hospitalized. This knowledge gap warrants attention for two reasons. First, early implementation of effective self-rehabilitation strategies during hospitalization could help establish beneficial training habits that continue after discharge. Second, understanding how goal setting influences unsupervised training during inpatient rehabilitation could guide the development of more effective self-rehabilitation protocols for the post-discharge period.

Based on these considerations, this study aimed to investigate whether goal setting immediately before a 15-min unsupervised reaching task affects behavioral performance and motivation in hospitalized patients with subacute stroke. We tested the following null hypotheses: (1) there is no difference in behavioral performance, including total time spent in training and success rate of reaching task between goal-setting and control groups; (2) there is no difference in self-reported motivation between groups.

## Methods

### Study design

This study used a single-blind, stratified randomized controlled trial where participants were allocated to either a goal-setting group or a control group. Participants were blinded to the study’s specific hypothesis regarding goal-setting effects on behavioral performance, as they were informed that the study aimed to investigate factors affecting rehabilitation motivation but were unaware that behavioral performance metrics were outcome measures. The study protocol was approved by the ethics committees of Hamamatsu University School of Medicine (approval number: 18–271) and Hamamatsu City Rehabilitation Hospital (approval number: 18–33), adhering to the 1964 Declaration of Helsinki and its later amendments. This trial was registered with the University Hospital Medical Information Network Clinical Trials Registry (UMIN000036156) on March 11, 2019 (https://center6.umin.ac.jp/cgi-open-bin/ctr/ctr_view.cgi?recptno=R000041168). All participants provided written informed consent. Patients or the public were not involved in the design, conduct, reporting, or dissemination plans of this study.

### Participants

Participants were recruited between October 2019 and January 2022. The inclusion criteria were as follows: (1) stroke patients aged 20 years or older admitted to Hamamatsu City Rehabilitation Hospital; (2) Brunnstrom Recovery Stage (BRS) of IV or higher for the upper-extremity and fingers; (3) sufficient cognitive function to understand the study procedures and perform voluntary training; (4) ability to sit independently in a chair or wheelchair; (5) desire to recover upper limb or finger function; and (6) provision of written informed consent for study participation. We excluded patients with aphasia that would prevent understanding of study procedures or other cognitive impairments that potentially affect the performance of reaching task, such as unilateral spatial neglect and apraxia. The presence of unilateral spatial neglect and apraxia was assessed through clinical observation during daily activities, with formal testing using standardized tools when clinically indicated (e.g., Behavioral Inattention Test for neglect^25,26^; Standard Performance Test for Apraxia^27^. Two participants had cerebellar stroke documented in their medical records but demonstrated adequate motor coordination for study participation as evidenced by meeting functional inclusion criteria.

### Sample size

The sample size was determined based on Whitehead et al.‘s recommendations for pilot studies^28^. For pilot studies with medium standardized effect sizes (0.3 ≤ δ < 0.7), they recommend 15 participants per treatment arm^28^. We assumed a medium effect size (δ = 0.5) based on meta-analytic evidence from goal-setting interventions in rehabilitation^29^. Levack et al.‘s Cochrane review reported variable effect sizes: large effects on self-efficacy (SMD = 1.07) and small to medium effects on engagement in rehabilitation (SMD = 0.30)^29^. Given that our study examined both behavioral and psychological outcomes, we adopted a medium effect size as a conservative estimate between these documented ranges. Although Whitehead et al.‘s recommendation suggests 15 participants per arm would be sufficient, we recruited 25 participants per group (50 total) to ensure adequate power for detecting clinically meaningful differences in our outcomes. This conservative approach accounts for potential dropouts and the novelty of goal-setting interventions in unsupervised stroke rehabilitation.

### Experimental procedures

After obtaining informed consent, participants were randomly allocated to either the goal-setting group or control group. The allocation sequence was generated by an independent researcher using stratified block randomization based on sex and disability level (BRS). Stratification by sex was implemented because studies have shown sex differences in motor function in older adults^30,31^. Stratification by disability level was used to ensure balanced distribution of motor impairment severity between groups, as this factor could significantly influence reaching task performance. Subsequently, we assessed cognitive functions using the Mini-Mental State Examination (MMSE)^32,33^ depressive symptoms using the Geriatric Depression Scale Short Form (GDS-S)^34^ apathy using the Apathy Scale (AS)^35^ and self-esteem using the Rosenberg Self-Esteem Scale (RSES)^36^.

**Fig. 1:**
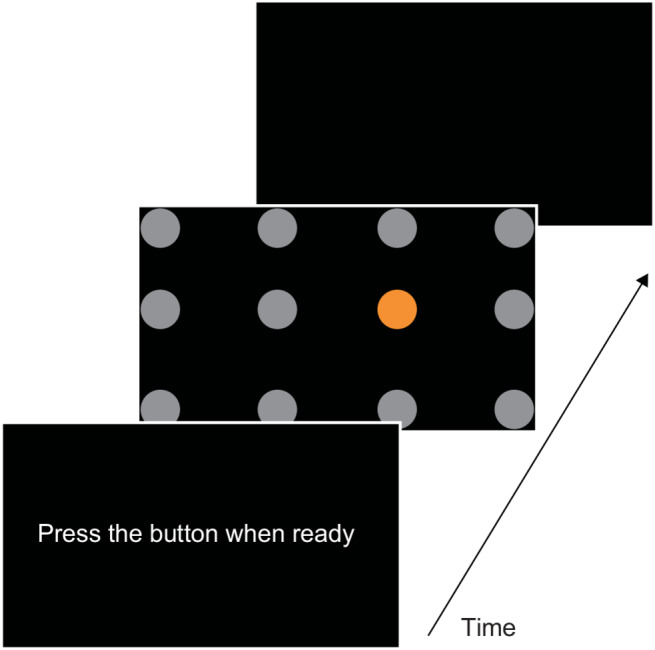
Schematic representation of the reaching task. The diagram illustrates the trial sequence used in the study. The screen displays 12 circular stimuli arranged in a 3 × 4 grid (one orange target circle among 11 gray distractor circles). The timeline depicts the sequence of a single trial: participants begin by pressing the “Press the button when ready” prompt, then respond by touching the orange target on the screen within the allocated time window (stimulus presentation time individually calibrated for each participant, median = 1000 ms), after which the sequence advances to the next trial.

On the experimental day, the initial 10 trials of reaching task (Fig. 1) were designed to ensure participants fully understood the task requirements. If a participant demonstrated difficulty understanding the task, additional explanations and demonstration trials were provided. Following this, all participants completed 30 practice trials to determine individualized task difficulty levels. During these trials, participants were instructed to perform the reaching movements as quickly and accurately as possible. We calculated the median reaction time for successfully hitting the target across these 30 practice trials for each participant. The stimulus presentation time for the unsupervised reaching task was then individually calibrated by rounding up the participant’s median reaction time. For example, if a participant’s median reaction time was 850 ms, the stimulus presentation time in the unsupervised task was set at 1000 ms; similarly, if the median reaction time was 3500 ms, the stimulus presentation time was set at 4000 ms. This individualized calibration process ensured that task difficulty was minimized across all participants. In fact, there was no significant difference in visual stimulus presentation time between the goal-setting group (median, 1000 ms; IQR, 1000–1000 ms) and the control group (median, 1000 ms; IQR, 1000–2000 ms) (U = 288.00, *p* = 0.766).

In the goal-setting group, participants received feedback with setting specific upper limb function goals using the SMART (Specific, Measurable, Achievable, Relevant, and Time-bound) framework^37^ from an occupational therapist, who had experience in stroke rehabilitation and had received training in the SMART goal-setting framework, while the control group received feedback without goal setting. Both groups then performed an unsupervised reaching task lasting up to 15 min. During the subsequent unsupervised phase, participants could take breaks or stop at any time. A break was defined as when the participant intended to resume training but temporarily stopped reaching activity. The task was terminated either when participants requested to stop or when the 15-minute period elapsed. The therapist monitored participants from an unobtrusive position to ensure safety. Although participants were informed about the study procedures, they were unaware that behavioral performance was an outcome measure.

Adverse events were defined as any untoward medical occurrence during the experimental session, including falls, excessive fatigue, cardiovascular symptoms, neurological deterioration, or any event requiring medical intervention or study discontinuation. Occupational therapists continuously monitored participants throughout all experimental procedures, with immediate documentation of any observed events.

### Goal setting

In the goal-setting group, the therapist first asked participants to identify personally meaningful goals related to their paretic upper limb function in daily activities. These goals were then refined using the SMART criteria³⁷ to ensure they were specific and measurable (e.g., achieving a certain degree of shoulder flexion), achievable within their rehabilitation period, and relevant to their recovery. For example, one participant who expressed a desire to resume driving worked with the therapist to develop the following SMART goal: “Achieve left hand participation in steering wheel operation (12–13 degrees rotation) by November.” This goal was Specific (steering participation), Measurable (degrees of rotation), Achievable (based on current BRS), Relevant (driving goal), and Time-bound (by November). The therapist then explained how the reaching task movements would develop the coordination needed for this functional goal. This structured goal-setting process took approximately 15 min and was conducted immediately before the unsupervised reaching task.

### Reaching task

All participants performed an unsupervised reaching task using their paretic hand. Schematic representation of the reaching task is shown in Fig. 1. Each trial started when the participant pressed a 7-cm diameter round plastic button (TouchPiko, Kokusai Dengyo Co., Ltd., Nagoya, Japan), which was placed in front of them, with their paretic hand. The task apparatus included a touch screen monitor (T232HL Abmjz, Acer Inc.) with a display area of 509 × 286 mm. Twelve circular stimuli (50 mm diameter) appeared on the screen arranged in a 3 × 4 grid, with one randomly positioned orange target among 11 gray circles. Participants were instructed to touch the orange target as quickly and accurately as possible within the stimulus presentation time, which ranged from 1 to 4 s based on their practice performance. After each response, feedback was displayed for 1.5 s, followed by a prompt to initiate the next trial. An incorrect response was recorded if participants touched a non-target stimulus or failed to respond within the time limit. Visual stimuli presentation and response recording were controlled by a custom-made program (Takei Scientific Instruments Co., LTD, Niigata, Japan) running on Windows OS.

### Outcome measures

The primary outcome measure was behavioral performance as assessed by the total times participated in and the success rate of reaching tasks during the unsupervised training lasting up to 15 min. The total time spent in unsupervised training was recorded as a measure of unsupervised training engagement. The success rate was calculated as the ratio between the number of successfully hitting the target and the total number of the target appearance during the unsupervised training phase. A high success rate indicates that a participant can accurately perform reaching movements to the targets. Although success rate was inadvertently omitted from the original trial registration, this measure represents a fundamental and clinically essential outcome for evaluating the quality of motor performance in goal-setting interventions and was therefore included as an outcome before data analysis. The secondary outcome was self-reported motivation for the reaching task, which was subjectively rated using a Visual Analog Scale immediately after the end of the task^38–41^. The possible score ranged from 0 (no motivation) to 100 (highest motivation), measured in millimeters on a 100-mm line.

### Statistical analysis

We compared demographic and clinical characteristics between the groups using two-tailed Mann-Whitney U tests for continuous variables and Fisher’s exact tests for categorical variables. For the primary analysis, we calculated the median values of total participation time (sec), accuracy (%), and motivation (mm) for each participant. As the Shapiro-Wilk test indicated non-normal distribution of the data, we used Mann-Whitney U tests to compare these outcomes between groups. Effect sizes were calculated using Cohen’s r, with values of 0.10–0.29, 0.30–0.49, and ≥ 0.50 indicating small, medium, and large effects, respectively^42^. Missing data were not included in the analysis. All analyses were performed using the Statistical Package for the Social Sciences software version 28.0 (IBM Corp., NY, USA). Statistical significance was set at *p* < 0.05. This study is reported in accordance with the CONSORT 2025 guidelines^43^ and the completed checklist is provided as Supplementary Table S1.

## Results

### Participants

**Fig. 2:**
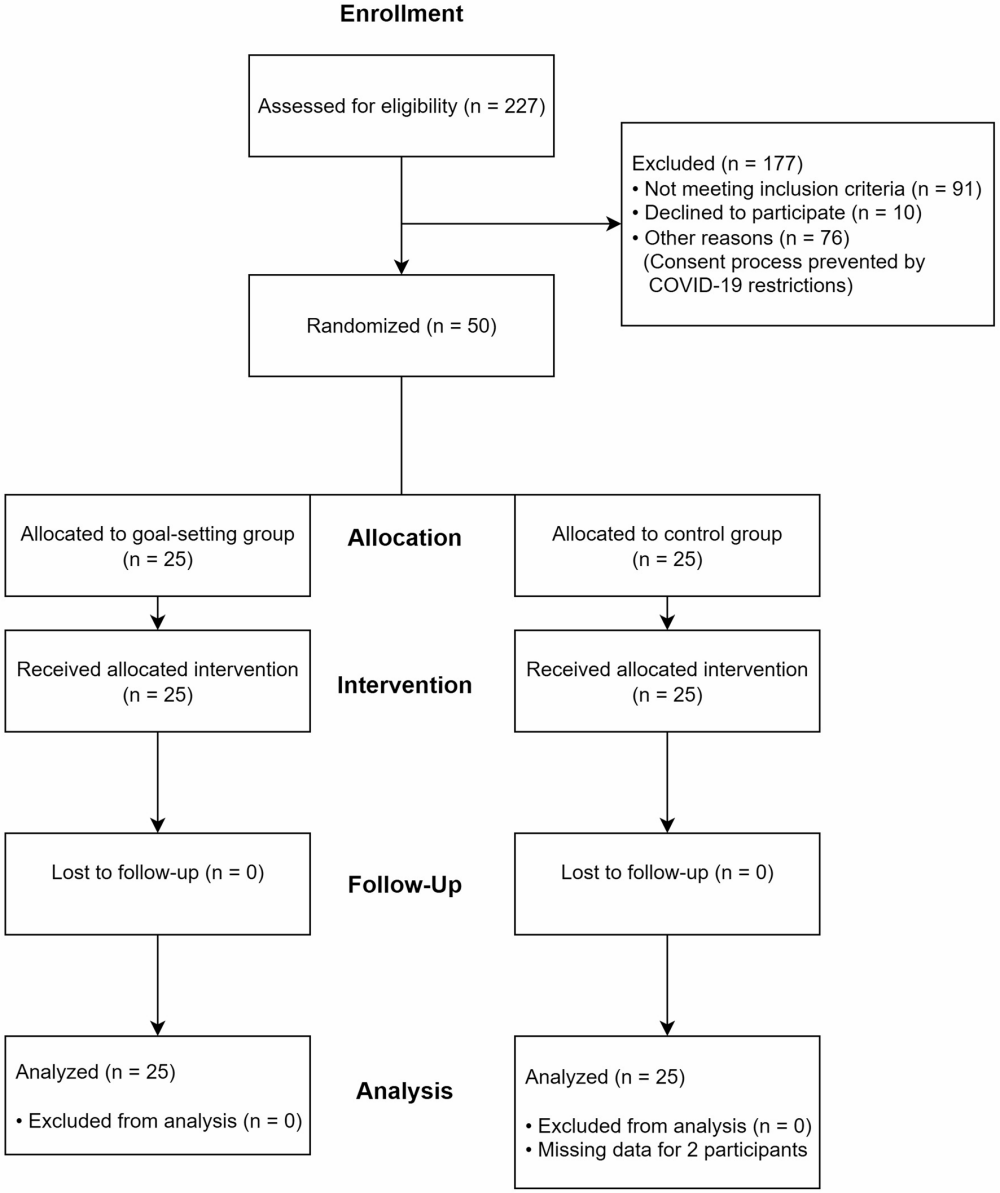
Participant flow through the study.

The flow diagram is presented in Fig. 2. A total of 227 participants were assessed for eligibility, of whom 177 were excluded: 91 did not meet inclusion criteria, 10 declined to participate, and 76 could not be approached due to COVID-19 restrictions preventing the consent process. Fifty patients were enrolled and randomized into two groups of 25 participants each. All participants completed the study protocol. No significant adverse events occurred during the study. Two participants in the control group had missing data: one participant had missing psychological assessment data (GDS-S, AS, and RSES scores) and another participant had missing self-reported motivation scores, both due to an administrative oversight during data collection. Analyses were conducted using available data for each outcome measure (*n* = 50 for behavioral outcomes, *n* = 49 for motivation scores. There were no significant differences between the two groups in terms of participant characteristics (age, sex) and clinical background at admission (e.g., side of motor paresis, BRS) (Table 1). Psychological measurements, including MMSE, GDS-S, AS, and RSES, also showed no significant differences between the groups (Table 1).

**Table 1:** Participants’ characteristics.

	All (*n* = 50)	Goal-setting group (*n* = 25)	Control group (*n* = 25)	Statistics
Sex, female/male	15/35	8/17	7/18	*p* = 1.000
Age	65.5(59, 71)	64 (57, 71.5)	66 (58, 71.5)	*p* = 0.763
Type of stroke, ischemic/hemorrhage	32/18	16/9	16/9	*p* = 1.000
Time since stroke (days)	67 (52, 91)	64 (52.5, 84.5)	70.0 (49.5, 92.5)	*p*=0.968
Side of motor paresis, left/right	27/23	14/11	13/12	*p* = 1.000
BRS	5 (5, 6)	5 (4.75, 6)	5 (5, 5.25)	*p* = 0.824
MMSE	29 (27, 30)	29 (28, 30)	28 (26.75, 30)	*p* = 0.176
GDS-S*	3 (1.5, 6)	3 (0, 5.25)	3.5 (2, 6)	*p* = 0.158
AS*	14(10, 17.5)	15 (10.25, 21.25)	13 (8.5, 17.5)	*p* = 0.464
RSES*	25 (23, 26)	25 (23.75, 26.25)	25 (23.5, 26)	*p* = 0.556

Values are presented as median (interquartile range) or number of participants. BRS = Brunnstrom Recovery Stage (range 1–6, higher scores indicate better motor recovery); MMSE = Mini-Mental State Examination (range 0–30, higher scores indicate better cognitive function); GDS-S = Geriatric Depression Scale Short Form (range 0–15, higher scores indicate more depressive symptoms); AS = Apathy Scale (range 0–42, higher scores indicate more severe apathy); RSES = Rosenberg Self-Esteem Scale (range 10–40, higher scores indicate higher self-esteem).

The asterisks indicate that one participant in the control group had missing data.

### Behavioral outcomes

**Fig. 3:**
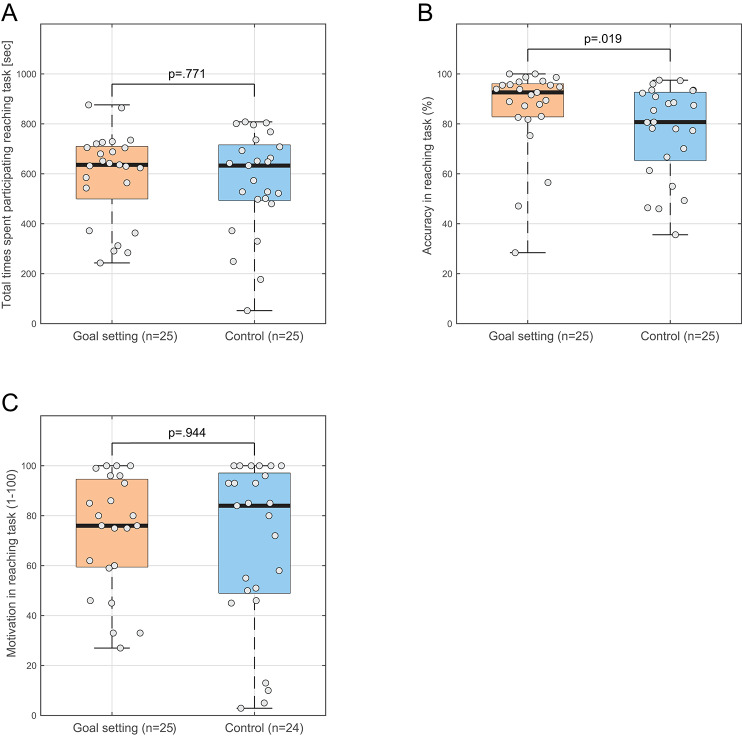
Behavioral and psychological outcomes comparing goal-setting and control groups. Box plots showing (**A**) total time spent in unsupervised training, (**B**) movement accuracy, and (**C**) self-reported motivation scores. The goal-setting group showed significantly higher accuracy compared to the control group (*p* = 0.019), while no significant differences were found in training time or self-reported motivation scores. Box plots display medians (horizontal lines), interquartile ranges (boxes), and individual data points (circles). One participant in the control group had missing data for the self-reported motivation scores.

The total time spent on the reaching task did not differ significantly between the goal-setting group (median, 636 s; IQR, 500–709) and the control group (median, 633 s; IQR, 494–715) (U = 297.5, *p* = 0.771, Cohen’s *r* = 0.041, Fig. 3A). In contrast, the goal-setting group demonstrated significantly higher success rate of reaching tasks (median, 92.6%; IQR, 82.9–96.0) compared to the control group (median, 80.7%; IQR, 65.4–92.6) (U = 192, *p* = 0.019, Fig. 3B), with a moderate effect size (Cohen’s *r* = 0.331). The median number of reaching trials performed during the 15-minute sessions was 181 (IQR, 136–216) in the goal-setting group and 187 (IQR, 164–212) in the control group (U = 290, *p* = 0.841).

### Psychological Outcomes

There was no significant difference in self-reported motivation scores between the goal-setting group (median, 76; IQR, 60–95) and the control group (median, 84; IQR, 49–97) (U = 296.5, *p* = 0.994, Cohen’s *r* = 0.010, Fig. 3C).

## Discussion

The present study investigated the effects of goal setting on behavioral performance and motivation during an unsupervised reaching task in hospitalized patients with subacute stroke. Regarding our null hypotheses, we failed to reject the null hypothesis for total training time and self-reported motivation, indicating no significant differences between groups for these outcomes. However, we rejected the null hypothesis for success rate of reaching tasks, demonstrating significantly higher accuracy in the goal-setting group compared to the control group with a medium effect size (Cohen’s *r* = 0.331). As there were no significant difference in task difficulty between the groups, the results of success rates indicated that goal-setting may promote accurate performance even during the unsupervised training. Given that repetitive task-specific practice has beneficial effects on upper-extremity function in stroke survivors^44^ our results suggest that goal-setting potentially enhances the quality of self-rehabilitation for acquiring accurate reaching movements with the paretic upper extremity.

These findings extend our understanding of goal setting effects in stroke rehabilitation in three ways. First, although previous studies have demonstrated the benefits of goal setting in supervised rehabilitation settings of brain-damaged patients^15,45–47^ to our knowledge, our study is the first to show its effectiveness in unsupervised training conditions. This distinction is crucial as self-rehabilitation programs become increasingly important in addressing the growing demand for stroke rehabilitation services⁵. Second, our results suggest that goal setting can help patients perform training task more accurately without continuous professional supervision, suggesting that well-structured goal setting may be a cost-effective strategy to promote movement quality during self-directed practice. Third, by demonstrating these effects in hospitalized patients with subacute stroke, our findings suggest that goal setting interventions can be effectively implemented during early rehabilitation, when patients are developing their training habits and routines.

The higher success rate of reaching tasks in the goal-setting group can be explained by several potential mechanisms. Most notably, the SMART framework used in our study helped participants establish specific, measurable goals related to their upper limb function, which were then explicitly linked to the reaching task requirements³⁷. This clear connection between personal goals and task performance may have enhanced participants’ motivation and/or attention to movement quality rather than just task completion^48,49^. Additionally, the goal-setting process might encourage participants to view each reaching movement as a meaningful step toward their rehabilitation goals, rather than simply as a repetitive exercise. This shift in perspective aligns with Social Cognitive Theory, which emphasizes how personal goal investment can enhance the cognitive regulation of performance^13,50^.

The absence of differences in training time between groups warrants careful consideration, as this was our primary outcome measure and a crucial indicator of engagement in self-rehabilitation. Several factors may explain our findings. First, the 15-minute time limit may have created a ceiling effect, as participants in both groups maintained relatively high engagement throughout the session (median time > 10 min). This suggests that the task parameters and environmental conditions might have been sufficiently engaging regardless of goal setting. Second, the timing of our goal-setting intervention—immediately before the task—may have been too brief to influence sustained engagement. Third, the structured hospital environment, where all participants were already engaged in regular rehabilitation programs, may have created a context that encouraged task completion regardless of explicit goal setting. Future studies should consider examining training time over multiple sessions and in more naturalistic settings (real-world clinical environments that reflect typical rehabilitation conditions, as opposed to highly controlled laboratory settings) to better understand how goal setting influences long-term engagement in self-rehabilitation.

The absence of between-group differences in self-reported motivation scores may reflect methodological limitations rather than a true lack of motivational effects. Our single-item visual analog scale assessment, while practical for clinical settings, may not have adequately captured the complex nature of motivation in stroke rehabilitation^22,41^. Additionally, the high motivation scores observed in both groups (medians > 75) suggest a possible ceiling effect, likely due to our inclusion of participants who were already actively engaged in rehabilitation programs. Future studies should consider employing more comprehensive motivation assessments. For instance, validated scales such as the Motivation in stroke patients for rehabilitation scale^40^ could provide more detailed insights into the motivational mechanisms underlying goal-setting effects. Alternatively, other factors such as attentional focus may have also contributed to improved performance as the mechanism of action for goal setting^48,49^.

Our findings have important implications for clinical practice in stroke rehabilitation. The more accurate reaching in the goal-setting group demonstrates that a brief, structured goal-setting session before self-rehabilitation can enhance movement quality without requiring additional supervision. This is particularly relevant as healthcare systems increasingly rely on self-rehabilitation programs to meet rehabilitation demands⁵. However, the limited effect on training duration suggests that optimizing patient engagement may require additional strategies, such as periodic goal reviews or technology-based monitoring⁷. Healthcare providers should consider goal setting as one component within a comprehensive approach to self-rehabilitation.

Several limitations of this study should be acknowledged. First, our study examined only the immediate effects of goal setting during a single session, leaving questions about its long-term impact on self-rehabilitation engagement and motor recovery. Second, we focused on a specific reaching task, which may not fully represent the variety of movements required in daily activities. Third, generalizability may be limited to healthcare systems with longer inpatient stays. Our participants had a median time since stroke of 67 days and were still hospitalized, differing from systems like the United States where patients are discharged much earlier. Forth, this study was designed prior to CONSORT 2025 guidelines, resulting in some methodological aspects that do not fully align with current reporting standards, such as limited patient and public involvement and post-hoc outcome specification. Finally, although not statistically significant, there was an imbalance in the side of motor paresis between groups, with 36% of participants in the goal-setting group having right-sided paresis compared to 56% in the control group (*p* = 0.256). This imbalance could potentially have influenced accuracy outcomes, as motor control and learning may differ between the dominant and non-dominant upper extremities during reaching tasks.

In conclusion, this study provides preliminary evidence that a single session of structured goal setting may improve movement accuracy during unsupervised training in hospitalized stroke patients. These findings suggest that goal setting could be a valuable strategy for increasing movement quality during self-rehabilitation, particularly in settings where continuous professional supervision is not feasible.

## Supplementary Information

Below is the link to the electronic supplementary material.

Supplementary Material 1

Supplementary Material 2

## Data Availability

All data generated in this study are included in the supplementary data file.
